# Standard Carotid Endarterectomy versus Carotid Artery Stenting with Closed-Cell Stent Design and Distal Embolic Protection: does the age matter?

**Published:** 2019-01-06

**Authors:** A Peluso, D Turchino, A Petrone, AM Giribono, R Bracale, L Del Guercio, UM Bracale

**Affiliations:** 1 Vascular Surgery Unit, Department of Public Health, University Federico II of Naples, Naples, Italy; 2 Department of Medicine and Health Science, University of Molise, Campobasso, Italy

**Keywords:** carotid artery stenosis, carotid endarterectomy, endovascular treatment, stent, embolic protection device

## Abstract

Carotid artery endarterectomy (CEA) is considered the gold standard for treatment of symptomatic and asymptomatic carotid disease. Carotid artery stenting (CAS) is a less invasive approach and therefore could be considered a viable alternative to CEA, especially in high-risk patients or those with relative contraindications to CEA (i.e. actinic stenosis, post-CEA restenosis, previous neck or tracheostomy surgery, contralateral laryngeal nerve paralysis, etc.).

**Methods:**

The aim of this study is to evaluate the short- and medium-term outcomes of CAS performed with a single type of closed-cell stent design and distal filter protection by comparing the procedure with CEA based upon 3 endpoints: overall survival rate, stroke free survival rate and restenosis free survival rate.

The same endpoints were also evaluated in 2 different age groups, more and less than 70 years, to show possible age-based differences on outcomes.

Among 105 patients (77 males, 28 females), 74 were submitted to CEA and 31 were subject to CAS.

In all cases the same self-expanding stent with closed-cell design (XACT Carotid Stent, Abbott Vascular) and the same distal embolic protection device (Emboshield NAV, Abbott Vascular) were employed.

**Results:**

At 12 months, no statistically significant difference was observed in overall survival rates (CEA 93.2% vs CAS 93.5%, p=0.967) and restenosis free survival rates (CEA 94.5% vs CAS 96.8%, p=0.662).

An increased stroke free survival rate was observed in the CEA group when compared to the CAS group (CEA 100.0% vs CAS 93.5%, p=0.028).

The age-based endpoints didn’t show any significant difference.

**Conclusion:**

These results suggest that CEA still remains the gold standard of treatment for carotid stenosis given its greater efficacy in the prevention of stroke CAS. However, CAS could be considered as an alternative treatment to CEA to be used in select cases only.

## I. INTRODUCTION

According to data from the World Health Organization (WHO), stroke is the third leading cause of death, the first major cause of physical disability, and the second cause of dementia^1^ in adults in the Western world.

Severe carotid stenosis is the cause of about 20% of all strokes and therefore early diagnosis of lesions and suitable treatment can reduce the disability and mortality associated with stroke.

A direct relationship exists between the rate of carotid artery stenosis and the risk of ipsilateral stroke^2,3^. Carotid revascularization by means of Carotid Endarterectomy (CEA) has been proven to be highly successful in reducing stroke incidence among patients with moderate-to-severe symptomatic carotid stenosis as well as amongst those with severe asymptomatic carotid stenosis^4^.

However, some Randomized Controlled Trials (RCTs) reported a greater incidence of myocardial infarction with CEA than with the CAS^5^ procedure. For this reason, CAS is increasingly being adopted as an alternative procedure to CEA in patients with carotid stenosis.

The aim of this study is to evaluate the outcomes associated with CAS carried out with a single type of closed-cell stent design and distal embolic protection device and to compare the results with those of the CEA procedure. Three endpoints were established and evaluated: overall survival rate, stroke free survival rate and restenosis free survival rate.

## II. METHODS

Between January 2013 and January 2016, we analyzed retrospectively 105 patients (77 males, 28 females) with a mean age of 69.5 years (SD±9.34) who had been treated at our institute for asymptomatic (62/105; 59%) and symptomatic (43/105; 41%) carotid stenosis.

All patients were evaluated for the presence of the following risk factors for carotid steno-obstruction disease: smoke, hypertension, dyslipidemia, diabetes, coronary artery disease (CAD), chronic renal failure requiring dialysis treatment, and presence of ipsilateral neurological symptoms during the last 6 months (fleeting amaurosis, TIA or minor stroke), contralateral occlusion (table 1).

All carotid stenoses were evaluated by a duplex scan with the ECST method followed by a CT scan or MRI exam^6–7^.

The entire cohort was divided into 2 groups according to the different carotid revascularization procedures employed: a CEA group and a CAS group.

Before showing possible age-based differences on outcomes, both groups were analyzed on age using T-student method and then divided into 2 further groups each: more and less than 70 years CAS group and more and less than 70 years CEA group.

### The CEA Group

CEA was performed under general anesthesia in 74/105 patients (70.5%). An intravenous bolus of sodium heparin was administered (50–100 UI/Kg) before vessel clamping.

A stump pressure method (SP) was used to evaluate the safety of carotid cross clamping, applying a temporary shunt to reduce the rate of cerebral ischemia when SP <40mmHg^8^. Standard CEA procedure was performed in 67/74 (90.54%) patients. A bovine pericardium patch (XenoSure) was applied in 14 cases (20.9%) while the arteriotomy was closed by direct running suture in 53 cases (79.1%). Application of a temporary shunt was necessary in 3 cases (4.47%) due to low stump pressure value (SP=32 mmHg; SP=22 mmHg; SP=14 mmHg).

An eversion CEA procedure was performed in 7/74 patients (9.45%) to correct the concomitant tortuosity of the internal carotid artery. All patients received only a low dose of ASA (100 mg/die) or clopidogrel (75 mg/die) to reduce risk of post-operative stroke and restenosis because due to a lack of evidence to support the use of dual antiplatelet or high dose aspirin therapy in patients undergoing CEA^9^.

### The CAS Group

CAS was performed in 31/105 patients (29.5%). Three days before, all patients received a low dose of ASA (100 mg/die) and clopidogrel (75 mg/die). Endovascular procedures were performed under local anesthesia and the common femoral artery was used for vascular access in all patients.

In only 2 cases (6.45%), after several unsuccessful attempts to catheterize the external carotid artery, was it necessary to convert the transfemoral access into a transcervical one.

An intravenous bolus of sodium heparin was used in all patients during the procedure (50–100 UI/Kg).

In all cases the same closed cell stent design was implanted (XACT Carotid Stent, Abbott Vascular) using the same distal embolic protection device (Emboshield NAV, Abbott Vascular) ^10^.

**Figure 1:**
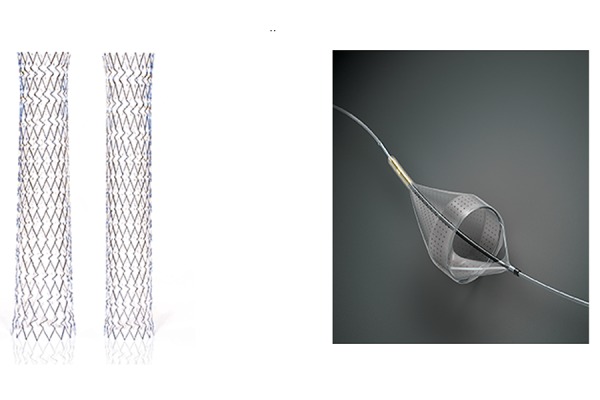
a) XACT Carotid Stent in both tapered and straight configurations; b) Emboshield NAV filtration element (available from https://www.vascular.abbott/int/index.html)

The XACT Carotid Stent, available in both tapered and straight configurations, is a self-expanding nitinol stent with a closed cell design and dense scaffolding to minimize tissue and plaque prolapse. Its targeted radial strength generated by variable cell sizes offers the strength suited to anatomy and lesion location (figure 1a).

The Emboshield NAV is a temporary percutaneous transluminal filtration system designed to capture embolic debris released during maneuvers within carotid arteries.

Its filtration element consists of a nylon membrane with an internal nitinol frame with two proximal entry ports and multiple distal perfusion pores for enhanced capture efficiency and flow preservation (figure 1b)^11^.

The mean stent diameter and length used on our patients was 8.0±1.3 mm and 38.4±3.7 mm, respectively.

Atropine (0.5–1 mg e.v.) was administered only in patients with a heart rate ≤50 bpm to prevent risk of bradycardia before stent release. Post-dilatation was performed with a Maverick XL Monorail Balloon Catheter.

All patients received dual antiplatelet therapy (clopidogrel 75 mg/die and ASA 100 mg/day) for at least 3 months after the procedure and then a single antiplatelet therapy (clopidogrel 75 mg/die or ASA 100 mg/die) indefinitely.

In patients with contralateral carotid occlusion, dual antiplatelet therapy was maintained lifelong. Follow-up included a duplex scan after 1, 3, 6 months and every 12 months thereafter.

## III. CLINICAL CHARACTERISTICS

Before performing statistical data analysis, all patients were assessed based upon their carotid stenosis risk factors: smoke, hypertension, dyslipidemia, type 1 and 2 diabetes, coronary artery disease (CAD), chronic renal failure requiring dialysis treatment, presence of ipsilateral neurological symptoms during the last 6 months (amaurosis fugax, TIA or minor stroke), contralateral occlusion (Table 1). As shown in Table 1, no single risk factor had a statistically significant difference in distribution in either group.

All data were analyzed with SPSS 20.0 (IBM) to generate Kaplan-Meier survival curves and Log Rank method was used to evaluate statistical significance.

## IV. RESULTS

During the follow-up period 7 deaths (CEA 6.7%, CAS 6.4%), 2 strokes (CEA 0%, CAS 6.4%), and 5 restenosis (CEA 5.4%, CAS 3.2%) occurred.

**Figure 2:**
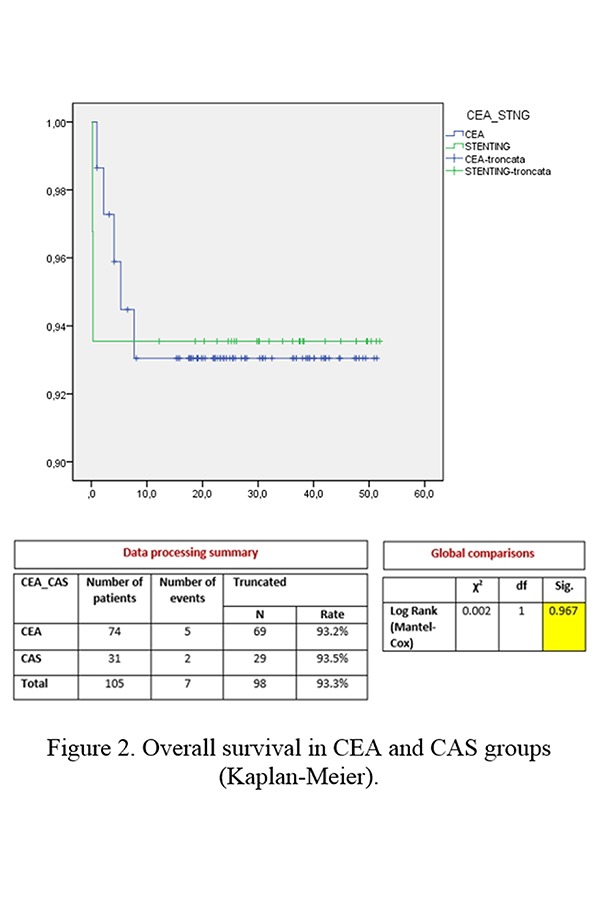
Overall survival in CEA and CAS groups (Kaplan-Meier).

No significant difference was found in the overall survival rate between the CEA and CAS groups (CEA 93.2% vs CAS 93.5%, p=0.967) (figure 2).

**Figure 3:**
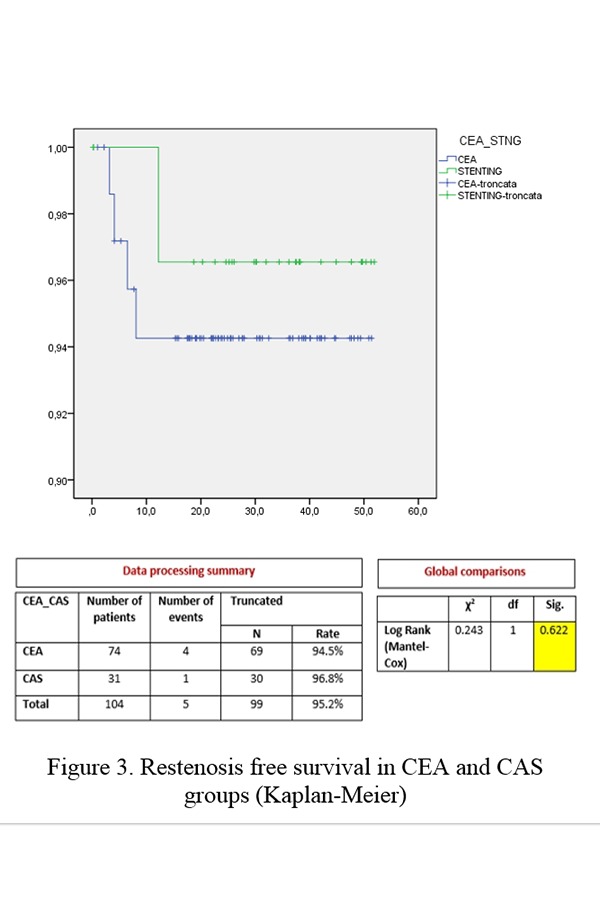
Restenosis free survival in CEA and CAS groups (Kaplan-Meier)

The restenosis free survival also showed no significant differences between the two groups (CEA 94.5% vs CAS 96.8%, p=0.622) (figure 3).

**Figure 4:**
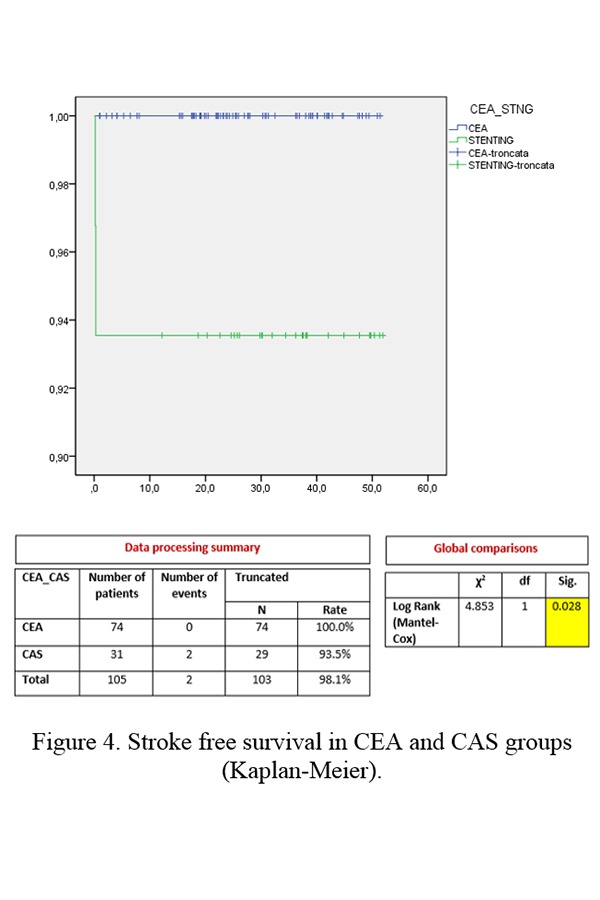
Stroke free survival in CEA and CAS groups (Kaplan-Meier).

On the contrary, the CEA group showed a higher stroke free survival rate than the CAS group (CEA 100.0% vs CAS 93.5%, p=0.028) (figure 4).

Furthermore, the only 2 strokes that occurred in the CAS group caused the death of the patients. An onset of latero-cervical hematoma requiring urgent surgical treatment a few hours after surgery took place in only 2 cases (2.7%) among the CEA group. No other complications occurred in either group.

The T-Student analysis on age didn’t show any significant difference between CEA and CAS groups (mean age: CEA 68.7±9.6 years vs CAS 71.5±8.3 years, p=0.493).

The age-based endpoints didn’t show any significant difference, as well (overall survival: more-70 years CEA 87.2% vs more-70 years CAS 90.5%, p>0.05; less-70 years CEA 100% vs less-70 years CAS 100%, not applicable; stroke free survival: more-70 years CEA 100% vs more-70 years CAS 90.5%, p>0.05; less-70 years CEA 100% vs less-70 years CAS 100%, not applicable; restenosis free survival: more-70 years CEA 97.4% vs more-70 years CAS 95.2%, p>0.05; less-70 years CEA 91.4% vs less-70 years CAS 100%, p>0.05).

## IV. DISCUSSION

The biggest problem in CAS procedure is embolic risk, especially during stent release. Certainly, the introduction of Embolic Protection Devices (EPDs) has significantly reduced this risk as demonstrated by the SAPPHIRE^12^, CREST^5^ trials and two systematic reviews^13–14^. However, not all types of commercially available carotid stents used in CAS procedures have the same impact on outcome. For example, the use of an open-cell stent design is associated with an increased 30-day stroke risk compared to a closed-cell stent design ^15^.

Recent CAS observational retrospective studies suggest that the use of closed-cell stents may be associated with lower stroke and death after stenting, compared with open cell-stents, particularly in symptomatic patients or in cases of vulnerable plaques. As well, the choice of a stent with a small free cell area can result in a significant decrease in post-procedural events^15^. Studies have concluded that the rates of periprocedural complications with CAS and CEA procedures were relatively lower in centers with experienced operators and surgeons who had verifiable good outcomes^16^. Over a 10-year period, both procedures were associated with stroke rates that were less than 7%^17^. Instead, at centers where interventional and surgical experience could not be verified several retrospective studies of clinical databases showed higher rates of periprocedural stroke or death after stenting than those reported in the CREST and ICSS^5^ trials.

Our own single center experience suggests that both CEA and CAS, when performed by experienced surgeons, are characterized by low prevalence of intraprocedural complications (CEA 2.7% vs 0% CAS).

According to SICVE (Italian Society of Vascular and Endovascular Surgery) guidelines, CAS is preferred to CEA whenever there is at least one of the following conditions in a patient: severe cardiac or pulmonary comorbidity, previous radiotherapy or neck surgery, tracheostomy, too high carotid bifurcation, contralateral laryngeal paralysis and/or post-CEA restenosis^18^. Also, surgeons prefer CAS over CEA on all patients with contralateral carotid occlusion due to their low cerebral tolerance to the carotid clamp^19–20^. In our analysis, however, the stroke free survival rate in the CEA group was higher than in the CAS group (CEA 100.0% vs CAS 93.5%, p=0.028).

In addition, the strokes in CAS group, which were attributable to the cerebral distribution area of the treated carotid artery, occurred no later than 10 days following procedure and all of them resulted in the patients’ death.

Although our study being monocentric with a small number of patients, and all data analyzed is retrospective, a single type of stent and protective device was however used. The results of our data lead us to two important considerations: (1) most ischemic events after a CAS procedure can occur within the periprocedural period (first 30-days from procedure) due to the fact that they are caused by an intrinsic embolic risk of the CAS procedure. Although such risk has been strongly reduced thanks to the introduction of several EPDs, it is still a significant risk; (2) although stroke prevalence in the CAS group was not particularly high (6.45%), in our experience its effects were nonetheless devastating, directly leading to patient death; (3) in our humble experience, elderly patients with significant carotid stenosis have a similar risk of postoperative complications as the non-elderly population. Therefore, the high risk aspects of CAS should always be taken into consideration when choosing the most suitable type of carotid revascularization procedure for the patient.

Based upon these 2 observations, we can conclude that CEA still remains the gold standard for treatment of carotid stenosis and that CAS with closed-cell stent can be considered as a viable alternative to CEA in selected cases only: severe cardiac and pulmonary comorbidity, previous radiotherapy or neck surgery, tracheostomy, post-CEA or post-CAS restenosis, very high stenosis or bifurcation, laryngeal paralysis, contralateral occlusion and restenosis. Furthermore, the procedures should be performed in centers with documented experience and training program operators control where surgeons can choose the most efficient technique according based upon their experience, the patient’s clinical condition and prevailing risk factors.

## 



## Figures and Tables

**Table 1:** distribution of risk factors for carotid stenosis in CEA and CAS groups.

Risk Factors	CAS (n=31)	CEA (n=74)	p<0,05
smoke	22 (70.96%)	43 (58.10%)	0.216
hypertension	23 (74.19%)	64 (86.48%)	0.127
dyslipidemia	17 (54.83%)	48 (64.86%)	0.335
diabetes	12 (38.70%)	26 (35.13%)	0.728
CAD (coronary artery disease)	9 (29.03%)	14 (18.91%)	0.253
dialysis	1 (3.22%)	5 (6.75%)	0.477
neurological symptoms	11 (35.48%)	32 (43.24%)	0.461
contralateral occlusion*	11 (35.48%)	0 (0%)	not applicable

* CAS was preferred to CEA whenever there was a contralateral occlusion.
