# Supplementary figures and images for: Standard Carotid Endarterectomy versus Carotid Artery Stenting with Closed-Cell Stent Design and Distal Embolic Protection: does the age matter?
Source: Transl Med UniSa. 2019 Jan 6;19:60–5. (PMC6581497)

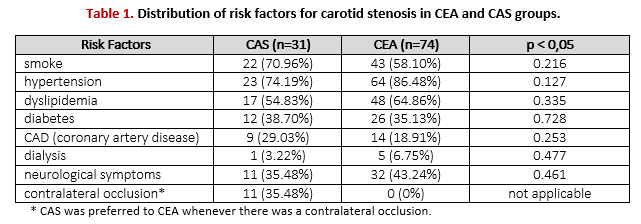


table 1: distribution of risk factors for carotid stenosis in CEA and CAS groups.

Supplement: Supplementary file 1 [file TM-19-060-s001.doc]
